# Is there a preferred time interval between gonadotropin-releasing hormone (GnRH) agonist trigger and oocyte retrieval in GnRH antagonist cycles? A retrospective cohort of planned fertility preservation cycles

**DOI:** 10.1007/s10815-024-03083-z

**Published:** 2024-03-16

**Authors:** Hizkiyahu Ranit, Herzberg Shmuel, Athavale Ahlad, Greenbaum Shirley, Harari Meny, Imbar Tal, Ben-Meir Assaf, Adler Lazarovits Chana, Bentov Yaakov, Esh-Broder Efrat, Hershko Klement Anat

**Affiliations:** 1 The IVF unit, Department of Obstetrics and Gynecology, Hadassah Mount Scopus-Hebrew University Medical Center, Jerusalem, 9112001 Israel; 2 The IVF unit, Department of Obstetrics and Gynecology, Hadassah Ein Kerem-Hebrew University Medical Center, Jerusalem, Israel; 3 Faculty of medicine, Hebrew university of Jerusalem, Jerusalem, Israel; 4 Faculty of medicine, Department of Obstetrics and Gynecology, Hebrew university of Jerusalem, Jerusalem, Israel

**Keywords:** Egg freezing, Oocyte retrieval, Gonadotropin releasing hormone, Hormone antagonist, Ovulation, Operative time

## Abstract

**Background:**

The ideal time frame between gonadotropin-releasing hormone (GnRH) agonist (GnRHa) trigger administration and oocyte retrieval in GnRH antagonist cycles has not been well studied. Our goal was to evaluate the effect of this time interval on oocyte yield and oocyte maturation rate in GnRH antagonist cycles designated for non-medical (“planned”) oocyte cryopreservation.

**Methods:**

We conducted a retrospective cohort study including patients who underwent elective fertility preservation, using the GnRH antagonist protocol and exclusively triggered by GnRH-agonist. We focused on the effect of the trigger-to-retrieval time interval on oocyte yield and maturation rate, while also incorporating age, body mass index (BMI), anti-Müllerian hormone (AMH) levels, basal Follicle-Stimulating Hormone (FSH) levels, as well as the type and dosage of gonadotropin FSH medication.

**Results:**

438 cycles were included. Trigger-to-retrieval time interval ranged from 32.03 to 39.92 h. The mean oocyte yield showed no statistically significant difference when comparing retrievals < 36 h (*n* = 240, 11.86 ± 8.6) to those triggered at ≥ 36 h (*n* = 198, 12.24 ± 7.73) (*P* = 0.6). Upon dividing the cohort into four-time quartiles, no significant differences in the number of retrieved oocytes were observed (*P* = 0.54). Multivariate regression analysis failed to reveal any significant associations between the interval and the aforementioned variables.

**Conclusions:**

The GnRHa trigger to oocyte retrieval interval range in our cohort did not significantly affect oocyte yield and maturation rate.

**Supplementary Information:**

The online version contains supplementary material available at 10.1007/s10815-024-03083-z.

## Background

The process of ovarian stimulation, oocyte retrieval and vitrification for the purpose of fertility preservation was initially introduced for oncological patients due to concerns about the harmful effects of gonadotoxic treatments. Following the experience gained with cryopreservation of oocytes and the subsequent removal of its experimental label by the American Society for Reproductive Medicine (ASRM), this procedure is now offered for various medical and social reasons [1, 2] .Fertility preservation for non-medical reasons, also called social, elective or planned fertility preservation, has gained popularity during the last few years and has increased significantly worldwide, with an 880% increase in the United States alone from 2010 to 2016 [3]. However, despite the growing use of oocyte vitrification for fertility preservation, it is still a relatively new technology with limited data available to understand its characteristics, safety, effectiveness and future consequences [4–6].

The GnRH antagonist protocol was first introduced as a shorter treatment duration compared to the long GnRH agonist protocol, with the advantage of avoiding estrogen deprivation symptoms [7]. Later on, the GnRH antagonist protocol was found to be even superior to the agonist protocol due to a substantial reduction in ovarian hyperstimulation syndrome (OHSS) [8], without reducing the live birth rates [9]. The advantage of short treatment duration, along with GnRHa as a trigger almost eliminating the risk of OHSS [10], has led the GnRH antagonist protocol to be a mainstay in fertility preservation for cancer patients [11, 12] and, later, in elective fertility preservation. In a retrospective study of 5289 patients from the Instituto Valenciano de Infertilidad clinics in Spain, 91% were treated by antagonist protocol [13]. The ideal time frame between GnRHa trigger administration and oocyte retrieval in GnRH antagonist cycles has not been well studied. Previous studies have examined different time frames while using human chorionic gonadotropin (HCG) for final oocyte maturation [14, 15]. Other studies included both HCG and GnRHa trigger in their analysis [16, 17]. The Euro-pean Society of Human Reproduction and Embryology (ESHRE) Working Group on Ultrasound in ART states that most authors recommend a 36 h interval between triggering and oocyte retrieval [18]. However, only one study from 2021 has investigated the time frame solely between GnRHa trigger and oocyte retrieval in intracytoplasmic sperm injection (ICSI) cycles and found a positive correlation between time interval and the number of oocytes collected, as well as the number of mature oocytes [19].

In light of the scant literature on one hand, and the need to investigate and characterize the procedure of elective fertility preservation on the other hand, the aim of our study was to further explore the effect of the time interval between GnRH agonist (GnRHa) trigger administration and oocyte retrieval on oocyte yield and oocyte maturation rate in GnRH antagonist cycles designated for planned oocyte cryopreservation. Our study hypothesis was that longer trigger-to-retrieval interval will result in a higher oocyte yield and a higher maturation rate.

## Materials and methods

We have conducted a retrospective cohort study utilizing data sourced from a tertiary hospital IVF clinics within the period spanning January 2021 to June 2023, encompassing both the Hadassah Mount Scopus (M.S) clinic and the Hadassah Ein Kerem (E.K) clinic. The study focused on patients who underwent planned fertility preservation (, employing the GnRH antagonist protocol and exclusively triggered by GnRH agonist (S.C. injection of 0.2 mg triptorelin ,Decapeptyl; Ferring, Germany). Medical fertility preservation cycles were excluded. Documentation of the precise trigger time in terms of hours and minutes was meticulously recorded within the charts by the nursing team. Noteworthy is the fact that due to our clinics’ standard procedure involving the administration of sedation during oocyte retrieval by an anesthesiologist, the computerized anesthesia chart inherently includes the surgical timetable, along with the pivotal “incision” moment, which directly corresponds to the actual puncture procedure. We calculated the time interval between trigger injection and the oocyte retrieval and analyzed its effect on oocyte yield and maturation rate, integrating age, BMI, AMH levels, gonadotropins FSH preparation type and dosage. The primary outcome measure was number of oocytes aspirated. Power analysis was based on Hershkop et al. [19], who reported a mean difference of roughly 3 oocytes in favor of a prolonged time interval (> 36 h). In order to demonstrate this difference with 80% power and α = 0.05, 152 cycles were required in each group (> versus ≤ 36 h). Other outcome variables quested were mature vitrified oocytes and the ratio between mature oocytes to aspirated oocytes (maturation rate).

### Ovarian stimulation protocols

Ovarian stimulation protocol included recombinant FSH (Gonal F: Merck Serono S.A., Darmstadt, Germany) or human menopausal gonadotropin (Menopur: Ferring Pharmaceuticals) or a recombinant FSH + recombinant LH medication (Pergoveris: Merck Serono S.A., Darmstadt, Germany), starting on cycle day 2–3. GnRH antagonist (Ganirelix [Orgalutran]: MSD, Petah-Tikva or Cetrorelix [Cetrotide]: Merck Serono S.A., Darmstadt, Germany) was added in a flexible multiple dose protocol. The standard daily starting dose of gonadotrophin was determined by the treating physician. When at least two follicles reached 17 mm or more in diameter, the GnRHa trigger was administered for final follicular maturation. GnRHa trigger was performed by S.C. injection of 0.2 mg triptorelin (Decapeptyl; Ferring, Germany).The trigger injection time is prescribed by the nursing team according to the expected procedural load: The E.K clinic nursing team starts prescribing a gradual timeframe at 9:30 PM while the M.S clinic team starts at 8:00 PM. All procedures in our institute are performed in the morning hours and all patients are admitted during the early morning hours. Due to the day to day variations in the procedural load, different time frames are created. The actual time interval was calculated and was utilized for the research. .

A computerized database including the following variables was established: age, BMI, basal FSH, AMH, AFC, gonadotropin formulation, total FSH dose, stimulation length, basal estradiol level and estradiol level at trigger, time interval length, number of oocytes retrieved and number of mature oocytes which were vitrified.

### Ethics

The study was approved by the local Institutional Review Board (0632-22HMO). Written informed consent was not required for this retrospective study.

### Data analysis

All analyses were performed using SPSS 23.0 (SPSS Inc., Chicago, IL). Univariate correlations were tested by Pearson correlation coefficient or ANOVA for the following variables: age at cycle initiation, BMI, AMH, basal FSH, gonadotropin dosage and gonadotropin medication type. Normally distributed data were compared across study groups by t- test or by ANOVA, as required. Chi-square or Fisher’s exact test were used for comparing rates and proportions. Regression models were assembled for correcting correlations between the time interval and oocytes/ mature oocytes / fraction of mature oocytes, by integrating confounding factors, which were significantly correlated with the outcome measure in a univariate analysis. All *P*-values were tested as two-tailed and considered significant at < 0.05.

The effect of the interval between GnRHa trigger to oocyte retrieval was analyzed in two different ways. First, we compared two groups: less than 36 h, and 36 h or more [18, 20, 21]. Second, we split the cohort into 4 quartiles )by statistical software) in order to avoid assumptions and to maximize grouping equality: <35.22, 35.23–35.85, 35.86–36.35 and > 36.36 h, and analyzed the interval effect according to the 4 quartile groups.

## Results

Our cohort comprised 438 cycles. Of these, 246 (56.2%) were first cycles, 133 (30.4%) second, and 41 (9.4%) third. The remaining cycles were of higher order (18 cycles, 4.1%). Patients` mean age was 34.9 ± 3.39 years on retrieval day (Table 1) and close to a statistical significance when compared between treating units, though roughly 35 years in both (Sup. Table 1a).The interval between trigger and oocyte retrieval ranged from 32.03 to 39.92 h. A summary of the group characteristics is presented in Table 1 and in Supplementary Table 1a. As AFC was documented in only 188 cases, we did not integrate it into our models. We relied on the superiority, or at the very least non inferiority, of AMH as a marker for expected oocyte yield [22]. Ovarian stimulation involved recombinant FSH (rFSH) in 93 cycles (21.2%), rFSH + rLH in 247 cycles (56.4%), and highly purified human menopausal gonadotropins in the remaining 98 cycles (22.4%).

**Table 1:** Patients’ clinical characteristics presented by Mean ± SD and Median values

Parameter	Mean ± SD	Median
Age (Years)	34.90 ± 3.39	34.96
BMI (Kg/m²)	24.33 ± 5.00	23.09
AFC	13.72 ± 6.85	13.00
AMH (ng/ml	2.54 ± 2.21	1.90
Basal FSH (IU/l)	7.37 ± 4.82	6.70
Total FSH dose (IU)	2,887.75 ± 1295.59	2,697.00
E2 before egg retrieval (pmol/l)	11,722.19 ± 6714.84	10,369.31
Trigger and oocyte retrieval interval (hours)	35.81 ± 0.77	35.85
Number of oocytes derived	12.03 ± 8.23	10.00
Maturation rate	0.77 ± 0.19	0.8

**Fig. 1:**
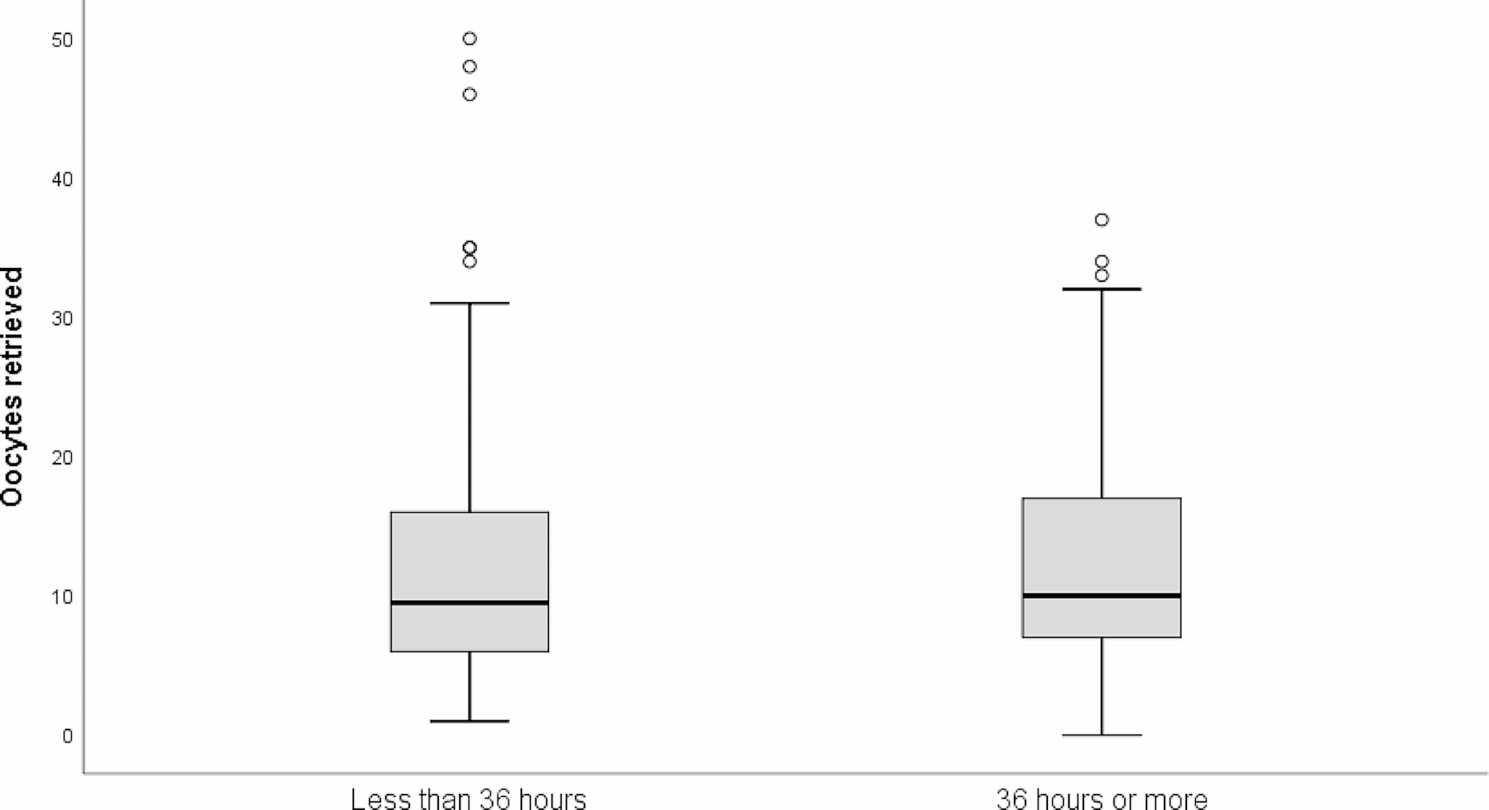
Box plot of retrieved oocytes by trigger to retrieval interval groups. *<36 h interval = 240 cycles, ≥ 36 h interval = 198 cycles

We did not observe a statistical difference between the mean number of retrieved oocytes while comparing the group undergoing retrieval less than 36 h after trigger (*N* = 240, mean ± SD: 11.86 ± 8.6) and the group triggered at ≥ 36 h (*N* = 198, mean ± SD: 12.24 ± 7.73), with a *p*-value of 0.6. The distribution of oocyte yield across different interval groups is depicted in Fig. 1. Similarly, after splitting the cohort into 4 quartiles (≤ 35.22 h *N* = 104, 35.23–35.85 h *N* = 115, 35.86–36.35 h = 105 and ≥ 36.36 h, *N* = 114), no significant variation in oocyte yield was observed (*P* = 0.5). (Supplementary Table 1b, Supplementary Fig. 1). The interval groups were not statistically different in terms of age (*P* = 0.4), AMH (*P* = 0.3), basal FSH (*P* = 0.6), cycle number (*P* = 0.6) and BMI (*P* = 0.5). The distribution of medication (gonadotropin) type was significantly different across interval groups (*P* < 0.01) and was treated in the regression model.

The following variables were significantly correlated to the number of oocytes retrieved and were therefore integrated into the model: age (*P* < 0.01), AMH (*P* < 0.01), basal FSH (*P* < 0.01), gonadotropin type (*P* < 0.01) and total FSH dose (*P* < 0.01). BMI was excluded from model (*P* = 0.17). Model and coefficients are presented in Table 2: after correcting for the aforementioned confounders, only age, AMH level and gonadotropin dose were significantly correlated with the number of oocytes retrieved. Consistent with expectations, increasing age showed a negative correlation, while AMH demonstrated a positive association. Interestingly, increased total FSH dose exhibited a negative correlation with the outcomes. In contrast, the time interval displayed no association with the outcome (*P* = 0.57). Upon restricting the model to the first cycle exclusively, the time interval exhibited once more no association with the number of retrieved oocytes (*P* = 0.69). However, both AMH (*P* < 0.001) and FSH dose (*P* = 0.02) remained significant predictors.

**Table 2:** Regression model for number of oocytes retrieved

Parameter		β	t	*P* value
Intercept		10.14	0.51	0.61
Age		-0.24	-2.07	0.04
Trigger to retrieval interval		0.3	0.56	0.57
AMH		1.59	7.90	< 0.001
Basal FSH		-0.17	-1.47	0.14
Total FSH dose		-0.001	-3.47	0.001
Gonadotropin type	Recombinant FSH	-0.38	-0.35	0.73
	HMG	-1.22	-1.20	0.23
	Recombinant FSH + Recombinant LH (reference group)	0		

The models were similarly executed for two other outcome measures: mature oocyte yield and the ratio of mature oocytes (Supplementary Tables 2 and 3). Once more, the time interval did not impact the outcomes of interest.

Four patients in our cohort did not yield any mature vitrified oocytes: one went through the procedure and no oocytes were detected in the aspirated fluid after an interval of 36.3 h. This patient presented a single leading follicle in the monitoring period. For the other three patients 1,8 and 13 eggs were retrieved but non were mature following 35.48, 36.47 and 35.3 h respectively.

## Discussion

The time interval between the ovulation triggering and oocyte retrieval is considered an important factor that contributes to the procedure success; it is crucial to perform the follicular puncture after LH surge has occurred in order to successfully collect the detached oocyte from the follicular fluid [23], and in order to allow the oocyte to accomplish its maturation [23]. It is clearly essential to perform adapt a time schedule that enables the oocyte retrieval surge previous to follicular rupture.

At the very beginning of IVF, in a report from 1982, hCG was given 36–38 h before performing laparoscopic oocyte retrieval [24]. However, according to other reports, the common practice was to administer hCG 32–36 h before oocyte retrieval in order to avoid cycle cancellation due to a spontaneous LH surge [14, 20]. Since then, and specifically after the incorporation of GnRHa which inhibits the spontaneous LH surge, several studies have investigated the optimal lag time between hCG administration and oocytes retrieval in GnRHa protocol, with inconsistent results. In a prospective study from 1994, Mansour et al. compared 3 interval groups: 35, 36 and 37 h. They found similar number of oocytes retrieved between all groups, but maturation rate was higher in the 36- and 37-hours groups compared to the 35 h (77.4%, 79.47% and 49.6%, respectively, *P* < 0.001) [20]. Later, in a prospective study by Bjercke et al., there was no significant difference between a 34 to a 38 h interval in terms of oocyte yield, number of embryos, embryo scores, implantation rate and pregnancy rate [23]. One year later, a larger study of 533 patients who were randomly allocated times for oocyte retrieval, with an interval range of 33–41 h, found no difference in the IVF outcomes- oocyte recovery rates, fertilization rates and pregnancy rates- between different interval groups (33 to < 36, 36 to < 38, 38 to < 41 h) [14]. Maturation rate was not evaluated [14]. A different point of view was that of Raziel and his colleagues, who have investigated the effect of prolonging the interval from 35.3 ± 0.7 h to 38.6 ± 1.2 h in patients with ≥ 47% immature oocytes in their previous cycle. They found a significant increase in maturation rate in the prolonged interval [25]. To summarize all those conflicting results, a meta-analysis was conducted in 2011, showing that in the longer time interval (> 36 h), oocyte maturation rate was higher (RR, 0.67; 95% CI, 0.62–0.73) than in the shorter interval (< 36 h) [21].

The treatment protocol, which was utilized in all aforementioned studies, as already specified, was long GnRH agonist with hCG for trigger. The GnRH antagonist protocol, using either an hCG trigger or a GnRHa trigger for final oocyte maturation was studied to much lesser extent, usually without distinguishing between the trigger types. Trigger-to-retrieval interval for antagonist cycles was adopted from former agonist cycles. In a retrospective study of 511 IVF/ICSI cycle, three different protocols were included (short agonist, long agonist and antagonist) with either hCG or GnRHa for ovulation triggering. The percentage of mature oocytes was significantly lower in the interval of 33.45–34.45 h and was stable between 35 and 38 h. Pregnancy rates were similar between interval groups. The study conclusion was that oocyte retrieval should be scheduled at least 35 h after triggering [16]. More recent study compared the trigger-to-retrieval interval in 4 different ovarian stimulation protocols. Again, different trigger types were included. According to this study, in order to retrieve more than 60% oocytes and more than 80% mature oocytes, trigger-to-retrieval interval should be delayed according to the stimulation type: mild stimulation protocol < GnRH antagonist protocol < short agonist protocol < long agonist protocol [17]. To the vet of our knowledge, the only study that explored the interval related solely to the GnRHa trigger was published by Hershkop et al. in 2021. In their study, 220 patients who underwent ICSI were divided unequally to four interval groups: 34.00-34.99, 35.00-35.99, 36.00-36.99 and from 37.00 and longer hours. The proportion of mature oocytes was similar between the groups [19].

In our study, we focused on antagonist protocol cycles which were exclusively triggered by GnRH agonist. As first described by Lanzone et al. in 1989 [26], and later was re-evaluated by Segal and Casper [27], GnRH agonist triggering results in an increase in serum LH and FSH, leading to final oocyte maturation. Therefore, GnRH agonist was found to be an effective alternative to hCG for ovulation triggering, while reducing the risk of OHSS [10]. Therefore, GnRH agonist triggering gained popularity and became the treatment of choice for ovulation triggering in fertility preservation cycles.

Our study population was unique – patients who elect to undergo planned fertility preservation, without any known infertility. This enabled us to lessen a possible influence of selection bias on the outcomes. We report no correlation between the interval and the outcome measures including number of oocytes retrieved, mature oocytes and maturation rate. Our results correspond with the only previous study in which ovulation was triggered solely by GnRHa, which found that the proportion of mature oocytes was similar between different interval groups [19].

As for the other variables that were investigated, we found that age was negatively associated with oocyte yield. Advanced age, especially above 37 years old, is a well-established contributing factor to a decline in the number of oocyte collected, and affects fertility treatment success rates including live birth rates [28–30]. This points again the significance of bringing forward the age in which elective fertility preservation is being performed, as already demonstrated by Cobo and her colleagues [31]. Contrary to age, AMH level was positively correlated with the number of retrieved oocytes. AMH, as an ovarian reserve marker, is well accepted predictor for number of oocytes retrieved and cycle cancellation [32]. In our study we also noticed an unexpected correlation between increased FSH dose and decreased number of oocytes collected. This could be attributed to the high dose that is given beforehand in patients expected to have poor response, according to their baseline AMH or AFC level. This association is reported in a regression model correcting for measurable confounders. Nonetheless, there are possibly unmeasurable confounders contributing to this finding. Such unmeasurable confounders can be the subject for future research. Interestingly, a recent study by Orvieto et al. they have investigated the effect of increasing gonadotropins dose in a patients` second cycle of elective/planned oocyte cryopreservation. They found that despite increased dose, 55.2% of patients will have less oocyte retrieved [33].

Our study has several limitations. First, its retrospective nature, which may explain lacking information regarding AMH levels for some of the patients. Second, some patients were contributing more than one cycle. Although this should not necessarily affect interval analysis, we performed a sub analysis for first cycle only demonstrating a lack of effect. Our study novelty arises from investigating a question that was rarely addressed before. Thanks to a computerized database, we were able to extract an exact time documentation for our analysis. The gathered population from two IVF clinics` has provided a sufficient sample size and homogenous as possible - these two centers are geographically close, with similar patients’ characteristics.

To summarize, in the new and growing era of planned fertility preservation, we were first, to the best of our knowledge, to investigate the role of a GnRHa trigger-to-retrieval interval on treatment outcomes. Lack of association may provide some reassurance for patients and the healthcare providers and allow more flexibility in scheduling operation time. Larger, randomized controlled studies are needed in order to further support our results.

## Conclusion

In GnRH antagonist cycles for planned fertility preservation, different time intervals (ranging 32.03 to 39.92 h) between GnRHa trigger to oocyte retrieval were not found to be associated with the number of oocytes retrieved or with oocyte maturation rate.

### Electronic supplementary material

Supplementary Material 1

## Data Availability

Upon request.

## Abbreviations

AMH: Anti mullerian hormone
BMI: Body mass index
FSH: Follicle-Stimulating Hormone
GnRH: Gonadotropin-releasing hormone
GnRHa: Gonadotropin-releasing hormone agonist
IVF: In vitro fertilization
