# Supplementary material for: Is there a preferred time interval between gonadotropin-releasing hormone (GnRH) agonist trigger and oocyte retrieval in GnRH antagonist cycles? A retrospective cohort of planned fertility preservation cycles
Source: J Assist Reprod Genet. 2024 Mar 16;41(6):1531–8. doi: 10.1007/s10815-024-03083-z (PMC11224053; doi:10.1007/s10815-024-03083-z)
Supplement: Supplementary file 1 — Supplementary Material 1 [file 10815_2024_3083_MOESM1_ESM.docx]

Sup Figure 1: Box plot of retrieved oocytes by trigger to retrieval time interval quartiles


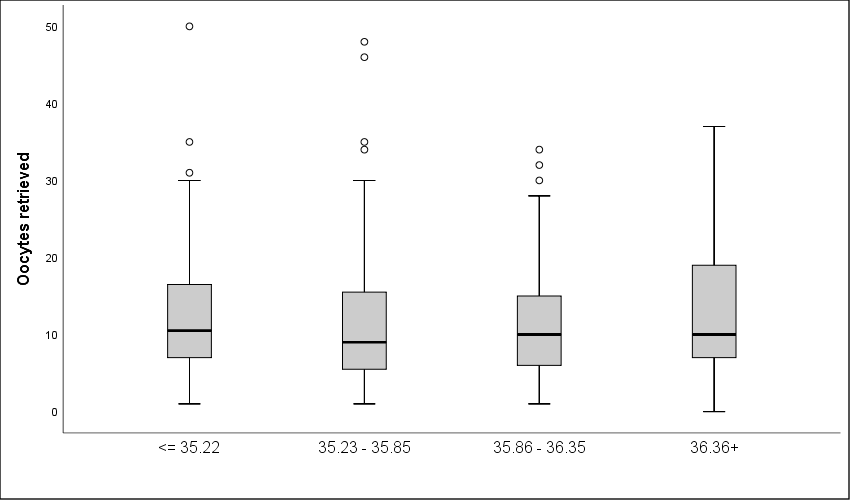


*≤35.22 hours N=104, 35.23-35.85 hours N=115, 35.86- 36.35 hours =105 and ≥36.36 hours, N=114

Sup.Table 1a. Baseline characteristics and oocyte yield according to treating unit (A,B)

| Unit | A | B | P value |
| --- | --- | --- | --- |
| Age | 34.6±3.5 | 35.1±3.2 | 0.053 |
| AMH | 2.4±2.2 | 2.6±2.2 | 0.2 |
| Cycel numner | 1.6±1.0 | 1.6±0.8 | 0.4 |
| Basal FSH | 7.6±6.1 | 7.1±3 | 0.2 |
| BMI | 24.4±5.3 | 24.3±4.6 | 0.8 |
| Oocytes retrieved | 11.7±8.8 | 12.3±7,6 | 0.5 |
| Mature oocytes | 9.3±9.5 | 9.0±6.9 | 0.4 |

Sup.Table 1b. Mean number of oocytes retrieved after splitting the cohort into trigger to retrieval time quartiles

| Interval (hours) | Number of cases | Mean number of oocytes retrieved± SD | 95% confidence interval for mean | | Mturation rate |
| --- | --- | --- | --- | --- | --- |
|  |  |  | Lower bound | Upper bound |  |
| <35.22 | 104 | 12.39 ± 0.83 | 10.74 | 14.05 | 0.8±0.16 |
| 35.23-35.85 | 115 | 11.75 ± 0.85 | 10.06 | 13.44 | 0.75±0.21 |
| 35.86- 36.35 | 105 | 11.23 ± 0.69 | 9.86 | 12.59 | 0.77±0.19 |
| >36.36 | 114 | 12.74 ± 0.75 | 11.25 | 14.23 | 0.8±0.2 |

Sup.Table 2. Regression model for number of mature oocytes retrieved

| Parameter | | β | t | P value |
| --- | --- | --- | --- | --- |
| Intercept | | 7.11 | 0.42 | 0.68 |
| Age | | -0.21 | -2.16 | 0.03 |
| Trigger to retrieval interval | | 0.28 | 0.62 | 0.54 |
| AMH | | 1.16 | 6.76 | <0.001 |
| Basal FSH | | -0.11 | -1.10 | 0.27 |
| Total FSH dose | | -0.001 | -3.33 | 0.001 |
| Gonadotropin type | Recombinant FSH | -0.35 | -0.38 | 0.71 |
|  | HMG | -0.63 | -0.73 | 0.47 |
|  | Recombinant FSH+ Recombinant LH (reference group) | 0 |  |  |

Sup.Table 3. Regression model for mature oocyte ratio

| Parameter | β | t | P value |
| --- | --- | --- | --- |
| Intercept | 1.32 | 2.89 | 0.004 |
| Basal FSH | 0.01 | 2.33 | 0.02 |
| Trigger and oocyte retrieval interval | -0.02 | -1.28 | 0.20 |

Sup.Table 4. Baseline characteristics and oocyte yield according by unit (A,B)

| Parameter | A | B | P value |
| --- | --- | --- | --- |
| Age | 34.6±3.5 | 35.1±3.2 | 0.053 |
| AMH | 2.4±2.2 | 2.6±2.2 | 0.2 |
| Cycel numner | 1.6±1.0 | 1.6±0.8 | 0.4 |
| Basal FSH | 7.6±6.1 | 7.1±3 | 0.2 |
| BMI | 24.4±5.3 | 24.3±4.6 | 0.8 |
| Oocytes retrieved | 11.7±8.8 | 12.3±7,6 | 0.5 |
| Mature oocytes | 9.3±9.5 | 9.0±6.9 | 0.4 |
